# Evolution of reproductive strategies in incipient multicellularity

**DOI:** 10.1098/rsif.2021.0716

**Published:** 2022-03-02

**Authors:** Yuanxiao Gao, Yuriy Pichugin, Chaitanya S. Gokhale, Arne Traulsen

**Affiliations:** ^1^ Department of Evolutionary Theory, Max Planck Institute for Evolutionary Biology, August-Thienemann-Str. 2, 24306 Plön, Germany; ^2^ Research Group for Theoretical Models of Eco-evolutionary Dynamics, Department of Evolutionary Theory, Max Planck Institute for Evolutionary Biology, August-Thienemann-Str. 2, 24306 Plön, Germany

**Keywords:** game theory, growth competition, life cycles

## Abstract

Multicellular organisms potentially show a large degree of diversity in reproductive strategies, producing offspring with varying sizes and compositions compared to their unicellular ancestors. In reality, only a few of these reproductive strategies are prevalent. To understand why this could be the case, we develop a stage-structured population model to probe the evolutionary growth advantages of reproductive strategies in incipient multicellular organisms. The performance of reproductive strategies is evaluated by the growth rates of the corresponding populations. We identify the optimal reproductive strategy, leading to the largest growth rate for a population. Considering the effects of organism size and cellular interaction, we found that distinct reproductive strategies could perform uniquely or equally well under different conditions. If a single reproductive strategy is optimal, it is binary splitting, dividing into two parts. Our results show that organism size and cellular interaction can play crucial roles in shaping reproductive strategies in nascent multicellularity. Our model sheds light on understanding the mechanism driving the evolution of reproductive strategies in incipient multicellularity. Beyond multicellularity, our results imply that a crucial factor in the evolution of unicellular species’ reproductive strategies is organism size.

## 1. Introduction

The evolution of multicellularity is viewed as a major evolutionary transition and it has occurred repeatedly across prokaryotes and eukaryotes [1–6]. With an increase in organism size, phenotypically heterogeneous organisms emerged through cell differentiation [2,7,8]. Reproductive modes of multicellular organisms may change with organism size and composition. In principle, multicellular organisms could reproduce multiple offspring with distinct cell numbers and organism composition—in contrast to their unicellular ancestors [9–13]. The number of possible reproductive modes rapidly increases with organism size. For example, for an organism containing three cells, two reproductive strategies are possible: split into three single-celled newborn organisms (1 + 1 + 1) or into a single-celled plus a two-celled newborn organism (2 + 1). For an organism containing 10 cells, there are 41 such reproductive strategies, and for a 20-celled organism, there are 626 reproductive strategies. However, only a few reproductive strategies dominate the tree of life. Some prominent examples abound, such as binary fission producing two single-celled organisms, multiple fission producing many single-celled organisms simultaneously [14–16], fragmentation reproducing many-celled propagules [13] and a special bottleneck reproductive strategy, a multicellular organism producing a single-celled newborn organism repeatedly [2,17,18].

The origin and the evolution of reproductive strategies are not well understood. Only a few reproductive strategies have been considered in previous work. The fragmentation mode of producing many-celled propagules has been investigated, in order to understand cell death in yeast [19] or to understand the advantages of multicellular life cycles experiencing a unicellular stage [10,17]. Previous work has examined the mechanism of life cycle transition from the unicellular stage to the multicellular stage. However, the underlying reproductive strategies are still unknown [20]. Recent work has also investigated mixed reproductive strategies [11,12], in which the fragmentation mode of an organism is not pre-determined, but selected by natural selection from all fragmentation modes. A subset of reproductive strategies with equal-sized offspring have been investigated in communities with cooperative interactions and deleterious mutations [21]. The majority of the literature is focused on the reproductive strategies of homogeneous organisms composed of identical cells. We have recently considered phenotypically heterogeneous organisms [9], but cellular interactions were restricted to linear frequency dependence and we ignored the impact of the organism size. Therefore, it is still unclear how organism size and cellular interaction, together, can shape reproductive strategies.

Organism size confers various advantages to organisms [22,23], such as avoiding predators [24,25], or incentivizing the division of labour [22,26]. Meanwhile, organism size can inhibit growth for different reasons, such as competition for space [19] or light [25]. Organism size can also affect reproductive strategies as early as nascent multicellularity [13,19,27,28]. Field observations are ambiguous about the effects of organism size [29–34]. Here, we consider a broad scope of size effects that can increase, decrease or not change the growth of heterogeneous organisms.

Previous studies have shown that cellular interactions can change reproductive modes [13,23,28]. For example, a new phenotype with a higher death rate leads to a reproductive mode of producing propagules among yeast *Saccharomyces cerevisiae* [13]. Phenotypically heterogeneous organisms could feature diverse cellular interaction forms. Here, we study cellular interaction that depends on a minimum threshold of a specific phenotype of an organism. This cellular interaction form has frequently been observed in nature. For example, in response to nitrogen depletion, cyanobacteria differentiate one heterocyst per 10 to 20 vegetative cells [15,35]. In the genus *Volvox*, along with the germ–soma differentiation [26], 1–20 germ line cells are produced among 500 and 42 000 somatic cells [36].

Thus, the size and composition could affect growth in phenotypically heterogeneous multicellular organisms. We develop a theoretical model to address the evolution of reproductive strategies considering the effects of organism size and thresholds for the number of different cell types. The size effects could increase or decrease organism growth, with the organism growing faster when the cell number of a particular phenotype reaches a given threshold. Organisms in a population share one common reproductive strategy while populations differ in reproductive strategies. Reproductive strategies thus compete with each other via population growth rates. The optimal reproductive strategy maximizes the population growth rate. We found that reproductive strategies can coexist or dominate others under different conditions. The uniquely optimal reproductive strategy always produces two offspring units.

## 2. Model

**Figure 1.**
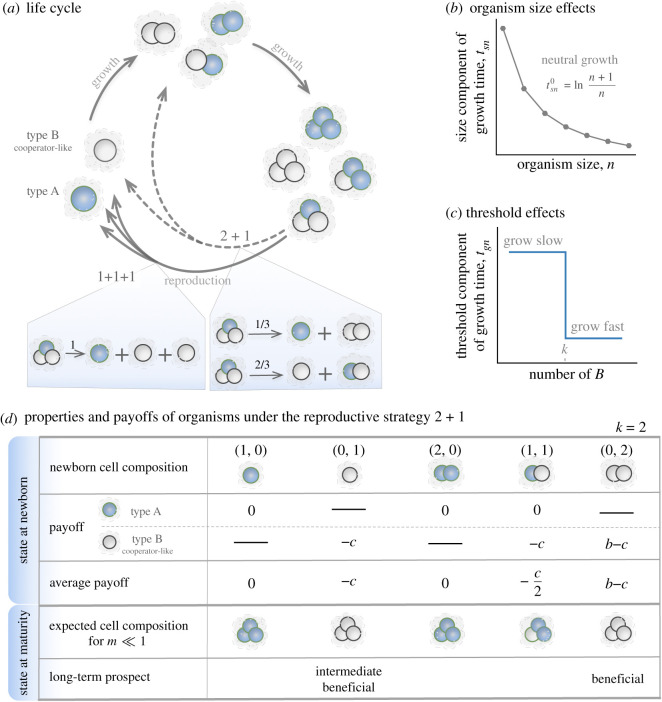
Illustration of a life cycle and the effects of size and threshold. (*a*) Example of life cycles with maturity size *N* = 3. Organisms with different cell compositions at each size stage are illustrated. Two reproductive strategies are shown: 1 + 1 + 1 and 2 + 1. In the shaded area, we show the probabilities of producing different newborn organisms from the mature organism (2, 1) under 1 + 1 + 1 and 2 + 1, respectively (see electronic supplementary material, appendix S1, for the calculation). (*b*) The organism size *n* affects the growth time of organisms. The grey dots show the neutral condition, where organisms of all sizes have the same growth rate. (*c*) Threshold effects on the growth time of organisms. In an organism when the number of *B* cells *n*_*B*_ exceeds the contribution threshold *k*, the threshold component of growth time *t*_*gn*_ decreases as in a volunteer dilemma game; see the main text. (*d*) An example of a population’s newborn organisms and their payoffs under threshold effects. We show the five possible newborn organisms of the population with reproductive strategy 2 + 1. The maturity size *N* = 3. The payoff of each cell in an organism and the average payoffs of organisms are given for *k* = 2. The expected cell composition describes an organism’s cell composition at maturity for *m* ≪ 1. Long-term prospect classifies fast-growing newborn organisms into ‘beneficial’ and ‘intermediately beneficial’; see the main text.

We consider multiple populations in which organisms grow and fragment into smaller pieces (figure 1*a*). The organisms in each population have a unique reproductive strategy. For example, a population with maturity size *N* = 3 must have the reproductive strategy either 1 + 1 + 1 or 2 + 1. In a population with 2 + 1, mature organisms with three cells produce a single-celled newborn organism and a two-celled newborn organism. The reproductive strategy determines the organism size at which an organism is born and at which size it is mature and reproduces. For the reproductive strategy *n*_1_ + *n*_2_ + · · · + *n*_*M*_, newborn organisms have cell number *n*_*i*_ (*i* = 1, …, *M*) and maturity size $N=\sum_{i=1}^{M}n_{i}$. We order offspring by size, such that *n*_1_ ≥ *n*_2_ ≥ · · · ≥ *n*_*M*_. We consider organisms consisting of two cell types: type *A* and type *B*. Newborn organisms may differ in their size and composition in a population. For example, a population with reproductive strategy 2 + 1 can have five types of newborn organisms: (1, 0), (0, 1), (2, 0), (1, 1) and (0, 2), where (*n*_*A*_, *n*_*B*_) shows the number of type *A* cells and type *B* cells in an organism (figure 1*d*). Each organism grows incrementally by one cell at a time. During each increment, a cell is selected to divide, and two daughter cells are produced. Each daughter cell can switch to another phenotype independently with a cell-type switching probability, which is typically *m* = 0.01 in our model (we also explore higher values later). After reaching their maturity size *N*, organisms reproduce via random fragmentation in terms of organism composition. The probabilities of forming different newborn organisms are calculated in electronic supplementary material, appendix S1. The newborn organism follows the same life cycle, growing from newborn to mature (see figure 1*a*).

We assume that organisms in populations grow independently without density dependence. Thus, populations follow exponential growth [37]. The population growth rate *λ*, depending on the number of offspring and the growth time of organisms [9,38], can be calculated as in electronic supplementary material, appendix S2. Since we assume no cell death, the number of offspring of each organism is constant, depending on its reproductive strategy. For example, with the reproductive strategy 2 + 1, organisms produce two offspring after reproduction. Thus, the population growth rate is determined by the time required for the newborns to mature. Assuming instantaneous reproduction, the growth time of an organism is then the sum of cell increment time $\sum t_{n}$, where *t*_*n*_ is the time for organisms growing from size *n* to (*n* + 1). The growth time depends on the cell size at the newborn stage and the cell size at the maturity stage. The time *t*_*n*_ is determined by the organism size and composition as

2.1 $$t_{n}=t_{sn}\times t_{gn},$$

where *t*_*sn*_ and *t*_*gn*_ are the size component and the threshold component of *t*_*n*_. Next, we discuss how we model *t*_*sn*_ and *t*_*gn*_.

**Figure 2.**
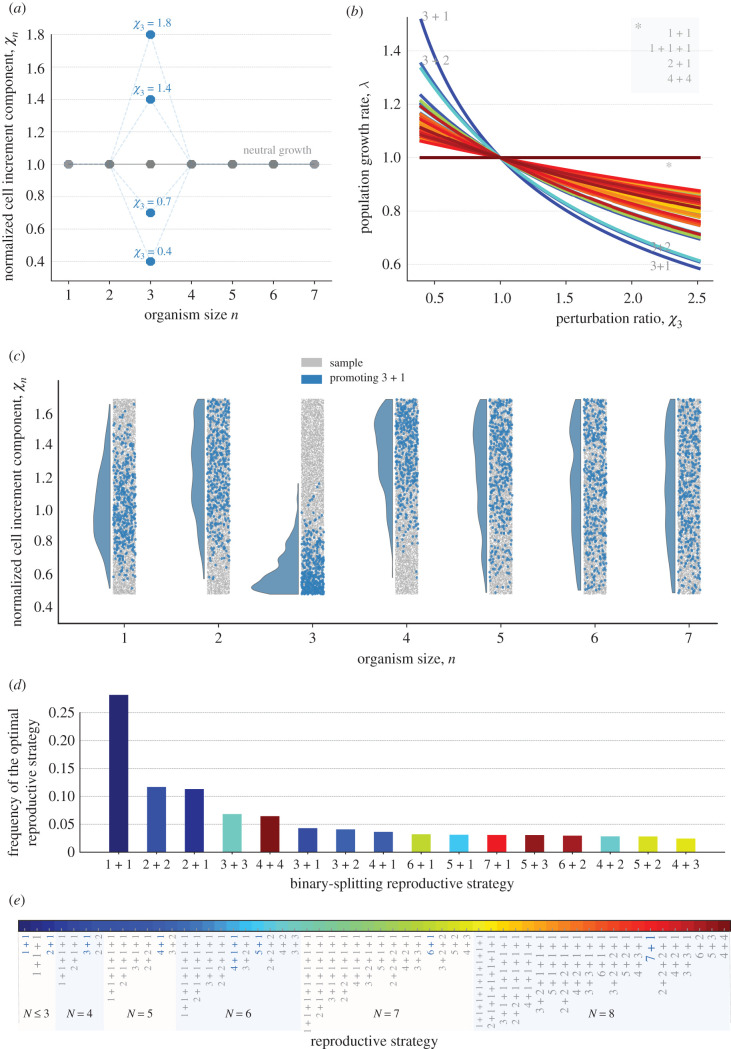
Binary-splitting reproductive strategies are uniquely optimal under the effects of size. (*a*) A diagram of perturbations at size *n* = 3. Grey dots are the conditions for neutral population growth *χ*_*n*_ = 1. Blue dots are the perturbed values at size 3 with different strength. (*b*) The growth rates of populations with different reproductive strategies under perturbations at size *n* = 3. The asterisk shows the unaffected reproductive strategies which continue to perform equally well. (*c*) The distribution of *χ*_*n*_ that promote the reproductive strategy 3 + 1 (in blue) among all samples (in grey). *χ*_*n*_ are drawn randomly from a uniform distribution, where *χ*_*n*_ = 0.5, …, 1.5. A sequence of [*χ*_1_, …, *χ*_7_] is randomly chosen at a time and the optimal reproductive strategy for it is identified. Ten thousand such sequences are investigated in total. Most notable, perturbations promoting 3 + 1 tend to have small values of *χ*_3_. (*d*) The frequency of observed optimal reproductive strategies under size effects. (*e*) The reproductive strategies that have been investigated for the maturity size *N* ≤ 8. The reproductive strategies highlighted in bold blue letters are the optimal ones under a single perturbation *n* = 1, …, 7.

The size component *t*_*sn*_ depends only on the cell number *n* = *n*_*A*_ + *n*_*B*_ of an organism during growth, but not on *n*_*A*_ or *n*_*B*_ individually. Under the neutral condition $t_{sn}^{0}=\gamma \ln(n+1)/n$, the doubling time of the organism size is independent of the organism size [9]. Thus, organisms of all sizes have the same growth rate (see figure 1*b*). Without loss of generality, we choose *γ* = 1 and treat $t_{sn}^{0}$ as a reference case. To analyse size effects beyond the neutral condition, we screen a large number of values of *t*_*sn*_ around the neutral condition ($t_{sn}^{0}$) (see figure 2*a*). We call $\chi_{n}=t_{sn}/t_{sn}^{0}$ normalized cell increment components, where *n* = 1, …, *N*. By perturbing all normalized cell increment components *χ*_*n*_ for *n* = 1, …, 7, we can capture all possible effects of size. For *χ*_*n*_ = 1, we recover the neutral condition.

The threshold component *t*_*gn*_ is determined by the number of the cell type of interest. Without loss of generality, we choose the type *B*. We assume the type *B* providing a benefit to the organism while bearing an individual cost. An organism grows faster if the number of its type *B* cells meets a given threshold *k* (figure 1*c*). There are many methods to construct such a compositional threshold effect. Here, we choose a volunteer dilemma game [39]. Consider an organism consisting of *n* cells with *n*_*A*_ type *A* cells and *n*_*B*_ type *B* cells. When *n*_*B*_ meets a contribution threshold *k*, each cell of the organism gets a benefit *b* from an increased organism growth. Each *B* cell pays an individual cost *c* and *A* cells pay no costs (figure 1*d*):

2.2 $$\begin{array}{rl} & P_{A}(n_{B})=\left\{ \begin{array}{cc} b & n_{B}\geq k\\ 0 & n_{B}<k \end{array}\right.\\ \mathrm{and}\; & P_{B}(n_{B})=P_{A}(n_{B})-c. \end{array}\}$$

These payoffs affect the division probability of the two types, i.e. which type is more likely to divide within an organism:

2.3 $$\begin{array}{rl} & p_{A}=\frac{n_{A}e^{wP_{A}}}{n_{A}e^{wP_{A}}+n_{B}e^{wP_{B}}}\\ \mathrm{and}\; & p_{B}=\frac{n_{B}e^{wP_{B}}}{n_{A}e^{wP_{A}}+n_{B}e^{wP_{B}}}, \end{array}\}$$

where *p*_*A*_ and *p*_*B*_ are the division probabilities for *A* cells and *B* cells, respectively, and *w* is the intensity of selection [40]. The threshold component *t*_*gn*_ is the inverse of average fitness of the organism:

2.4 $$t_{gn}={\left(\frac{n_{A}e^{wP_{A}}+n_{B}e^{wP_{B}}}{n_{A}+n_{B}}\right)}^{-1}.$$

To analyse such threshold effects, we will vary the contribution threshold value *k*.

## 3. Results

### 3.1. The effects of organism sizes on reproductive strategies

To focus on size effects only, we first assume no threshold effect, *w* = 0. We investigate size effects by perturbing a single normalized cell increment component *χ*_*n*_, starting from a fully neutral condition *χ*_*n*_ = 1, where *n* = 1, …, 7 (figure 2*a*). If the organisms of a population are going through a perturbed state at size *n*, i.e. $n_{M}\leq n\leq N=\sum n_{i}$, then its reproductive strategy (*n*_1_ + *n*_2_ + · · · + *n*_*M*_) can deviate from the one under the neutral condition. Since the population growth rate is inversely proportional to growth time, a perturbation is either advantageous (*χ*_*n*_ < 1, *λ* > 1) or disadvantageous (*χ*_*n*_ > 1, *λ* < 1) for population growth. A reproductive strategy is referred to as being promoted (suppressed) when its population growth rate is greater (smaller) than the neutral growth rate 1. A single advantageous perturbation (*χ*_*n*_ < 1) promotes the reproductive strategy of any population with organisms going through the state *n* of the perturbation, i.e. the strategies are satisfying *n*_*M*_ ≤ *n* ≤ *N* (figure 2*b*). The performance of reproductive strategies is unaffected when their populations’ organisms do not go through the size under perturbations, i.e. *n* < *n*_*M*_ or *n* > *N*. A single adverse perturbation *χ*_*n*_ > 1 suppresses reproductive strategies that satisfy *n*_*M*_ ≤ *n* ≤ *N*. Among these affected populations, we found that the reproductive strategy *n* + 1 is most affected by perturbations at size *n*. Since the population with reproductive strategy *n* + 1 contains *n*-celled newborn organisms, which mature at size *n* + 1, its growth time depends on *χ*_*n*_. Therefore, under the condition of *χ*_*n*_ < 1 and *χ*_*k*_ = 1 (*k* ≠ *n*, *k* = 1, …, 7), the reproductive strategy *n* + 1 is uniquely optimal. Under the condition of *χ*_*n*_ > 1, the reproductive strategy *n* + 1 is most suppressed (see figure 2*b*). Analogous to the reproductive strategy *n* + 1, the reproductive strategy *n* + 2 is the second most affected reproductive strategy. Similarly, for the rest of the reproductive strategies, their population composition determines whether the growth rates are affected or not. The growth rates then determine the performance of reproductive strategies.

We can analyse all possible size effects by combining single perturbations for *n* = 1, …, 7, which forms a discrete size function with respect to *n*. As each *χ*_*n*_ is independently perturbed from the neutral condition, the discrete function can cover all possible shapes. We investigate the general features of size functions that promote a given reproductive strategy, but ignore the specific shape of the size functions. As expected, we observed that the populations of optimal reproductive strategies contain organisms that mostly go through sizes with smaller *χ*_*n*_. This is illustrated in figure 2*c* and an analytical proof is given in electronic supplementary material, appendix S3, for reproductive strategies with *N* ≤ 3. We found that only the binary-splitting reproductive strategy (producing two offspring) can be uniquely optimal (see figure 2*d*; electronic supplementary material, appendix S4, for the analytical proof). Intuitively, this is because the fastest-growing newborn organisms in a population with a multiple-splitting reproductive strategy can also be found in another population with a binary-splitting reproductive strategy. For example, the population growth rate of 2 + 1 + 1 cannot be greater than that of 1 + 1 and 2 + 2 at the same time. Additionally, 1 + 1 is the most frequently observed reproductive strategy in binary-splitting reproductive strategies (figure 2*d*) because 1 + 1 is the only reproductive strategy that depends on a single cell increment component *χ*_1_. Therefore, for a randomly chosen *χ*_*n*_ (*n* = 1, …, 7), 1 + 1 has a higher probability of being optimal compared to other strategies. Generally, reproductive strategies have lower chances to be optimal when binary splitting makes organisms go through many cell increment stages.

### 3.2. The effects of thresholds on reproductive strategies

To investigate threshold effects exclusively, we assume the size effect to be neutral: *χ*_*n*_ = 1, such that $t_{sn}=t_{sn}^{0}$. For *b* − *c* = 0, cells of type *B* would never lead to any growth advantage; thus we focus on *b* − *c* > 0. With a threshold at size *k*, newborn organisms of a population with *n*_*B*_ ≥ *k* have larger payoffs and thus have shorter growth time; see equations (2.2) and (2.4). The growth of different newborn organisms determines the population growth rate. For example, consider all possible newborn organisms in the population with the reproductive strategy 2 + 1: (1, 0), (0, 1), (2, 0), (1, 1) and (0, 2) (see figure 1*d*). With the contribution threshold *k* = 2, (0, 2) grows fastest as it has two *B* cells. (0, 1) is the second-fastest-growing newborn organism as it most likely gains benefits by producing a second *B* cell during growth. (1, 0), (1, 1) and (2, 0) grow relatively slowly because they are less likely to produce at least two *B* cells during growth. For convenience, we refer to newborn organisms in a population as ‘beneficial’ if *n*_*B*_ ≥ *k* and ‘intermediate beneficial’ if *n*_*A*_ = 0, *n*_*B*_ < *k* and *N* > *k*, where *N* is maturity size. All other newborn organisms are unlikely to reap the benefits of cooperation before maturity. The growth rate of a population depends primarily on its beneficial newborn organisms and secondly on its intermediate beneficial newborn organisms. Moreover, among all beneficial newborn organisms, the one with exactly *k* type *B* cells has the largest payoff. Thus, they have growing advantages under no or low cell-type switching probability. For a low cell-type switching probability, e.g. *m* = 0.01, homogeneous newborn organisms are more abundant than heterogeneous ones. In the long run, we expect that populations mostly contain homogeneous newborn organisms.

**Figure 3.**
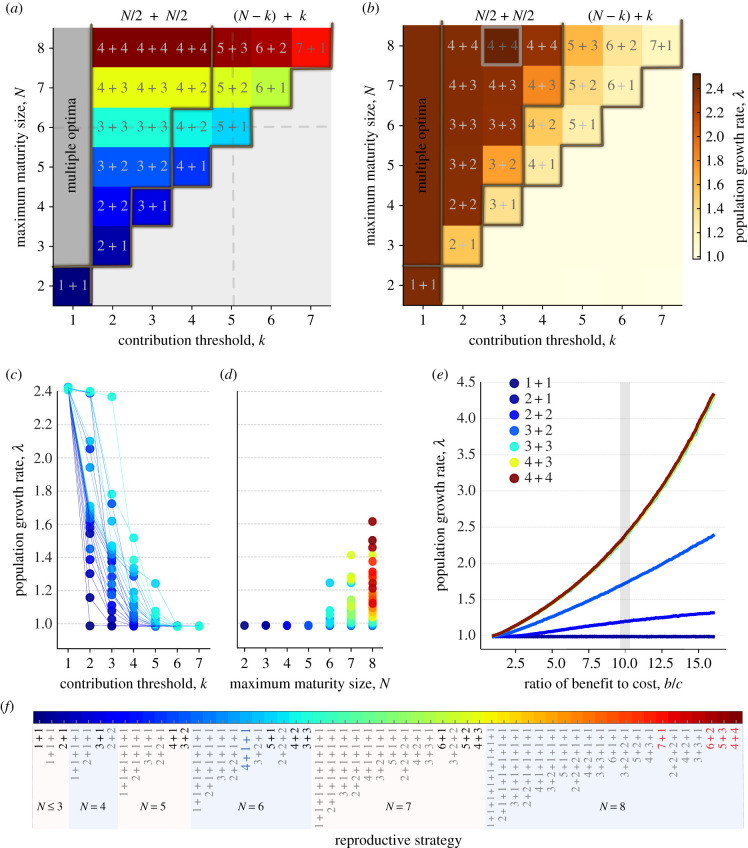
Binary-splitting reproductive strategies are uniquely optimal for threshold effects with *k* > 1. (*a*) The optimal reproductive strategies across contribution threshold *k* (*k* < 8) and maturity size *N* (*N* ≤ 8). The grey lines (in *a*,*b*) are the boundaries between multiple optimal reproductive strategies (at *k* = 1), symmetric binary-splitting reproductive strategies, asymmetric binary-splitting reproductive strategies and those that never meet the threshold. The grey dashed lines indicate the parameter space where we investigated the population growth rate of each reproductive strategy in (*c*,*d*). (*b*) The population growth rates of the optimal reproductive strategies in (*a*). The highlighted parameter set with *N* = 8 and *k* = 3 is investigated in more detail in (*e*). (*c*) Population growth rates of different reproductive strategies with *N* ≤ 6 are shown across different contribution threshold *k*. (*d*) Population growth rates of different reproductive strategies under contribution threshold *k* = 5 are shown across different maturity size *N* ≤ 8. (*e*) The growth rates of populations with symmetric binary-splitting reproductive strategy are shown across to varying ratios of benefit to cost. (*f* ) The reproductive strategies that have been investigated for *k* ≤ 7 and *N* ≤ 8. The optimal reproduction modes that appear in (*a*) are highlighted in black. The uniquely optimal reproductive strategies under the threshold effect for *k* = ≤7 and *N* ≤ 8 are highlighted in bold and red. Parameters for all panels: *w* = 0.1, *b* = 10, *c* = 1 and *m* = 0.01.

For threshold effects, the uniquely optimal reproductive strategies are binary splitting at the maximum maturity size: 4 + 4, 5 + 3, 6 + 2 and 7 + 1 (figure 3*a*). The optimal reproductive strategies can be classified into three categories: multiple optima, symmetric binary splitting (*N*/2) + (*N*/2) (or ((*N* + 1)/2) + ((*N* − 1)/2)) and asymmetric binary splitting with a *k*-celled newborn organism (*N* − *k*) + *k*. When *k* = 1, multiple reproductive strategies are optimal at the same time (see figure 3*a*–*c*). Since every population contains beneficial newborn organisms, the performances of different reproductive strategies are similar. As *k* increases, the symmetric binary-splitting reproductive strategies (*N*/2) + (*N*/2) (or ((*N* + 1)/2) + ((*N* − 1)/2)) are optimal for 1 < *k* ≤ (1/2)*N* (see figure 3*a*,*b*). Newborn organisms with size equal to or greater than *k* have growth advantages; thus, intuitively (*N*/2) + (*N*/2) and *k* + (*k* + 1) should have the same performance in population growth. However, we found that only (*N*/2) + (*N*/2) (or ((*N* + 1)/2) + ((*N* − 1)/2)) is optimal. The intrinsic composition of the population and the effects of cell-type switching probability *m* = 0.01 determine the results. To understand the growth advantages of the symmetric binary-splitting reproductive strategies with the maximal maturity size, we take 4 + 4 and 3 + 3 at *k* = 3 as an example. For *k* = 3, the population of 4 + 4 contains the beneficial newborn organisms (1, 3) and (0, 4). The population of 3 + 3 only contains beneficial newborn organisms (0, 3). When a cell-type switching event happens during growth, (0, 4) produces a beneficial newborn organism (1, 3), while (0, 3) reproduces a non-beneficial newborn organism (1, 2). Populations with larger maturity sizes are less affected by the cell-type switching probability as they contain multiple types of beneficial newborn organisms. Finally, when $\frac{1}{2}N<k<N$, the reproductive strategy (*N* − *k*) + *k* becomes optimal (see figure 2*a*). When $k>\frac{1}{2}N$, each individual can at most have one type of beneficial offspring organisms. Next, we explain why the optimal reproductive strategy is (*N* − *k*) + *k* rather than other reproductive strategies such as $k+\underset{N-k}{\underbrace{1+1\cdots +1}}$ and (*N* − *k* − 1) + *k* + 1. Because of *N* − *k* < *k*, organisms with *N* − *k* cells can only form intermediate beneficial newborn organisms—and only when they are pure *B* cells. Larger intermediate beneficial newborns grow faster than smaller ones. We take 3 + 1 + 1 and 3 + 2 for *k* = 3 as an example. 3 + 1 + 1 has the intermediate beneficial newborn organism (0, 1) and 3 + 2 has the intermediate beneficial newborn organism (0, 2). During organism growth, (0, 1) undergoes two cell increment stages with longer time (larger *t*_*gn*_ due to negative payoffs; see equations (2.4) and (2.2)), while (0, 2) only undergoes a single one. Thus, a population with the reproductive strategy 3 + 2 grows faster than one with 3 + 1 + 1.

Population growth rates decrease with increasing *k*, resulting from reducing the number of beneficial and intermediate beneficial newborn organisms. Especially when *k* ≥ *N*, no reproductive strategies will obtain the benefits of cooperation, and their populations grow slower due to the associated costs (see figure 3*a*,*b*). Increasing maturity size *N* increases population growth rates of the optimal reproductive strategies because the number of beneficial or intermediate beneficial newborn organisms increases. As expected, population growth rates also increase with the benefit to cost ratio (see figure 3*b*–*e*).

Additionally, we investigated the effect of varying *m* on the reproductive strategy for *N* = 8. We found that it is robust for the conclusion: the optimal reproductive strategy is binary splitting with maximum maturity size. At high *m* and high *k*, the asymmetric binary-splitting strategy 7 + 1 invades other asymmetric binary-splitting ones (electronic supplementary material, figure S2*a* in appendix S5). Since 7 + 1 can keep the largest number of *B* cells in a newborn organism, this organism could gain growth advantages for high cell-type switching probability. A higher cell-type switching probability increases the heterogeneity of organisms, especially for large organisms. This leads to fewer *B* cells in an organism—thus, the population growth rate generally decreases with increasing *m*; see electronic supplementary material, figure S2*b*. For some reproductive strategies, a change in the cell-type switching probability leads to complex effects on population growth (electronic supplementary material, figure S2*c*). This is the outcome of several factors: the cell-type switching probability *m*, the contribution threshold *k* and the structure of the population. We take the reproductive strategy 1 + 1 + 1 as an example to discuss the potential reason: when *k* = 2, the population has a lower growth rate when *m* = 0.5 because organisms cannot reach the threshold during their growth.

### 3.3. The combined effects of organism sizes and thresholds on reproductive strategies

**Figure 4.**
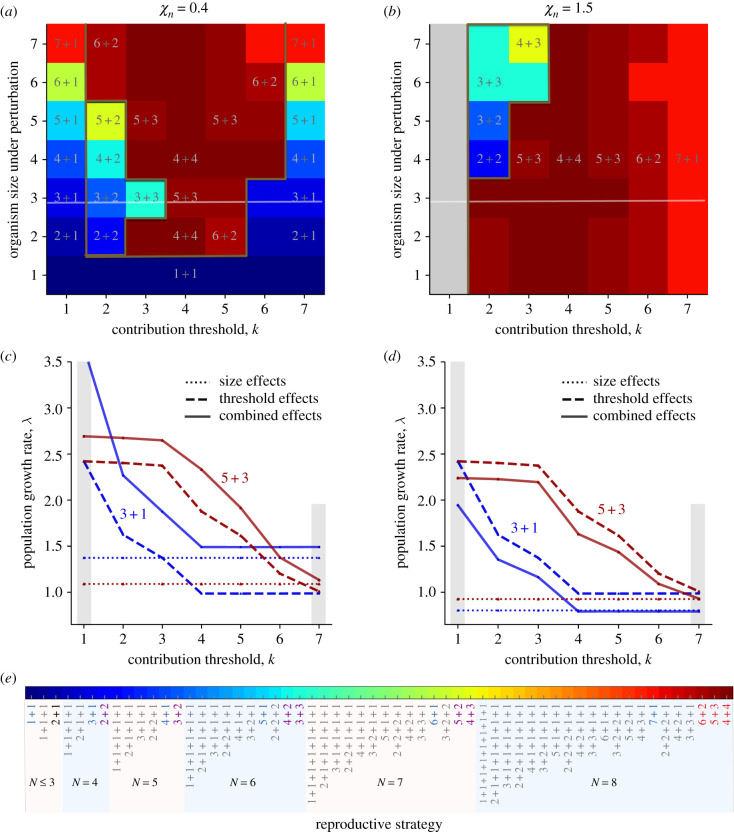
Binary-splitting reproductive strategies are uniquely optimal under the effects of size with a single perturbation and threshold. (*a*) Optimal reproductive strategies under the effects of single advantageous size perturbations and thresholds. (*b*) Optimal reproductive strategies under the effects of single adverse size perturbations and thresholds. In (*a*,*b*), the perturbation only occurs at a single size at a time. The dark brown lines indicate the boundaries of optimal reproductive strategies observed under a single perturbation, threshold effects and both. Note that 7 + 1 is uniquely optimal under either a single perturbation or threshold effects. Reproductive strategies are multiple-optimal under the grey area. The white lines indicate the parameter space where we investigate the population growth rate in (*c*,*d*). (*c*,*d*) The population growth rates of reproductive strategies 1 + 3 and 3 + 5 under the effects of a size perturbation at *n* = 3, threshold and both, respectively. In (*a*,*c*), *χ*_*n*_ = 0.4. In (*b*,*d*), *χ*_*n*_ = 1.5. (*e*) The reproductive strategies that have been investigated for *k* ≤ 7 and *N* ≤ 8. The reproductive strategies in blue are uniquely optimal under the size effect of a single perturbation. The reproductive strategies in red are uniquely optimal under the threshold effects. The reproductive strategies in purple are newly emerged uniquely optimal strategies under both a single perturbation and the threshold effect. Parameters for all panels: *w* = 0.1, *b* = 10, *c* = 1, *m* = 0.01.

Finally, we investigate the optimal reproductive strategies under size and threshold effects combined. For simplicity, we only consider the size effects in the form of a single perturbation. All binary-splitting reproductive strategies *n*_*i*_ + *n*_*j*_ can be uniquely optimal, where *n*_*i*_ and *n*_*j*_ are positive integers, and *n*_*i*_ + *n*_*j*_ ≤ *N* (figure 4*a*,*b*). With the combined effects of size and threshold, we found new optimal binary-splitting reproductive strategies that are neither optimal in the effects of single perturbation only nor for thresholds only, including 2 + 2, 3 + 2, 4 + 2, 5 + 2, 3 + 3 and 4 + 3. Furthermore, under a beneficial size perturbation, we found *n* + 1 (*n* = 1, …, 7) can be optimal both at small and large contribution threshold *k* (see figure 4*a*,*b*). This is due to the fact that the threshold effects lead to a similar performance of reproductive strategies either at small *k* and at large *k* (figure 3*b*). Therefore, for combined size and threshold effects, the size effect has a larger impact on the performance of reproductive strategies (see figure 4*a*,*c*). Consequently, the reproductive strategy *n* + 1 becomes optimal under an advantageous perturbation, where *n* = 1, …, 7. Newly emerged binary-splitting reproductive strategies have advantages for intermediate contribution thresholds *k*, suggesting that it is an outcome of the trade-off between the effect of size and threshold. For an adverse size perturbation, we found that the reproductive strategy *n* + 1 cannot be optimal (figure 4*b*), because the adverse size perturbation leads to poor performance of reproductive strategies that are influenced by the perturbation (figures 2*b* and 4*d*). The reproductive strategy 7 + 1 is an exception to this rule, as the threshold effect strongly influences it at *k* = 7. The optimal reproductive strategies observed are those that can obtain growth benefits from threshold effects and avoid the disadvantages from the adverse size effect. For example, 3 + 3 outcompetes 4 + 4 for *k* = 2 when a disadvantageous size perturbation occurs at *n* = 7. Both strategies can obtain growth advantages from threshold effects. However, adverse size perturbation decreases the population growth rate of 4 + 4, but it has no impact on 3 + 3. Thus the performance of reproductive strategies is the outcome of the trade-off between the effects of size and threshold. Our results suggest that all binary-splitting reproductive strategies can evolve under an appropriate choice of size effects (at a single size) and threshold effects.

## 4. Discussion

Numerous reproductive strategies are conceivable for multicellular organisms, but only recently more attention has been paid to the evolution of such reproductive strategies [9,11,12,20,41,42]. Here, we developed a theoretical model considering the effects of size and cell interaction on the evolution of reproductive strategies, impacting organism growth. We considered clonal organisms, in which cells stay together after cell division. An alternative way to form multicellular organisms is ‘coming together’, usually responding to adverse environments [2,3,42–44]—but here we entirely focus on ‘staying together’ instead, which typically leads to groups of identical cells when the probability of type switching is small. Our abstract model is loosely tied to biological examples and does not explicitly depict any specific organisms. For example, the cell-type switching between types can capture processes of phenotypic task-allocation in cells rather than epigenetic transformation between cells. Our model shows that only binary-splitting reproductive strategies (producing two offspring) can be uniquely optimal. All binary-splitting reproductive strategies can evolve under more complicated size effects or under threshold effects with a single perturbation. The result that uniquely optimal reproductive strategies are binary-splitting strategies under the size effects coincides with previous work [9,12]. Our results show that the uniquely optimal reproductive strategy is binary splitting. In turn, each binary-splitting reproductive strategy can be uniquely optimal. All binary-splitting reproductive strategies can evolve under threshold effects with a single size perturbation—suggesting that they can readily evolve under the combined effects of size and threshold.

The reproductive strategy *n* + 1 resembles organisms passing through a unicellular phase, which is a striking feature for most multicellular organisms [18]. Previously, organisms undergoing a unicellular stage have been attributed to reducing mutational load and regulating the cell conflict during growth [10,17]. Interestingly, our model shows that the reproductive strategy *n* + 1 can evolve under size or threshold effects. This indicates an alternative way for evolving organisms with a single cell bottleneck. Our models also show the conditions where multiple reproductive strategies can evolve simultaneously. Each reproductive strategy can evolve when the size effects are the same for each cell size (*χ*_*n*_ are the same) or when the threshold is low (for example, *k* = 1). Many reproductive strategies have beneficial newborn organisms (*n*_*B*_ ≥ *k*) when the threshold is low, leading to a large population growth rate. This result indicates that multiple reproductive strategies can coexist for an organism. Notably, the results resonate with the observation that one species can possess several reproductive strategies simultaneously in nature [14,15,45,46]. Cyanobacteria are one such example that have multiple reproductive strategies such as binary fission, budding and multiple fission.

In our model, we can choose a flexible impact of size on organism growth. We investigated the size effects by first investigating the effects of single size perturbations. Although single size perturbations seem unrealistic for organism growth in nature, they provide a theoretically convenient approach towards understanding size effects. Size effects that are present by combing single size perturbations could have positive, negative or neutral effects on growth at each cell increment. The model assumption corresponds to studies concerning size effect on growth [29–34]. The size perturbations used in our work allow a wide range of size functional forms to be investigated, including those studied in previous work [11,12,47]. If the size function is arbitrary (randomly choose each *χ*_*n*_), we found the frequently observed optimal reproductive strategy 1 + 1. The finding suggests that 1 + 1 is the best reproductive strategy unless explicit advantages exist for a larger organism. The size effects considered in our model are functions of cell numbers rather than the task allocation of each cell. Previous work on the division of labour has instead focused on continuous functions [27,48–50].

We delineated the threshold effect of cellular interactions in a multiplayer volunteer game given the utility of game theory in depicting biological interactions ranging from social foraging to cancer development [51–58]. We deliberately chose this game, as the threshold effect constitutes a strong nonlinearity that simpler games cannot capture [9]. The main difference between our work and previous investigations of volunteer games is that we primarily use the volunteer’s dilemma to capture cellular interactions [39,59]. In addition, we investigate the role of such interactions in the evolution of reproductive strategies but not the evolution of strategic behaviour in the underlying game. The cells cannot freely choose their actions in this game, and the two cell types switch at random.

We mainly investigated reproductive strategies with a small cell-type switching probability, *m* = 0.01. This focus assumed that cell-type switching is uncommon and mostly happens under environmental pressure in nature [3,60]. The low switching probability leads to a relatively homogeneous population, mainly containing homogeneous newborn organisms. If a population has beneficial (*n*_*B*_ ≥ *k*) (or intermediate beneficial (*n*_*A*_ = 0, *n*_*B*_ < *k* and *N* > *k*)) newborn organisms, then homogeneous beneficial (or intermediate beneficial) newborn organisms dominate the population. Although heterogeneous beneficial newborn organisms grow fastest, they are not most abundant. First, heterogeneous beneficial newborn organisms do not produce themselves fast after reproduction. The reason is that *A* cells have higher payoffs; thus, they produce more *A* cells during the growth of heterogeneous beneficial newborn organisms. When the organisms reproduce, they are unlikely to produce more than one heterogeneous beneficial newborn organism. Second, although heterogeneous beneficial newborn organisms can be replenished from the reproduction of beneficial newborn organisms (via cell-type switching), the replenishment is slow as beneficial newborn organisms have lower growth advantages. Thus, it is also theoretically intriguing to investigate reproductive strategies under high cell-type switching probability. Such an investigation shows that the conclusion is robust across cell-type switching probability parameter space: the optimal reproductive strategy is the binary-splitting one with maximum maturity size (electronic supplementary material, figure S2 in appendix S5). Although high cell-type switching probability mainly decreases the population growth rate, nevertheless it does not affect the relative performance of reproductive strategies. Within these binary-splitting strategies, the binary-splitting reproductive strategy producing the largest newborn invades others at high cell-type switching probability.

Our model investigated the competition of reproductive strategies via the growth rates of isolated populations. In many situations, this correlates with the ability to invade other populations—simply by outgrowing existing types. We can extend the model to a density-dependent population consisting of organisms with different reproductive strategies. Given the size and threshold effects, the invasive strategies would reflect the reproductive strategies observed in our model. Furthermore, our model considered the size effect via the number of cells in an organism, so the size function is discrete. In nature, some unicellular organisms grow by enlarging themselves rather than by cell divisions, such as *Stanieria*, *Pleurocapsa* and *Dermocarpella* [14]. Instead of binary fission, these unicellular organisms undergo multiple fission or asymmetrical multiple fission (analogous to $n+\underset{N-n}{\underbrace{1+1\cdots +1}}$) during the reproductive phase by a rapid succession of cell divisions. Since the size is typically a discrete variable for these unicellular organisms, our model implies that the maturity size plays an essential role in shaping the evolution of reproductive strategies for unicellular organisms. In the present model, the size is a discrete variable counting the number of cells in an organism and it is an important driver of the reproductive strategy. We expect that the size will also be important in models with continuous sizes. Overall, our work can provide insights into the evolution of reproductive strategies of complex multicellular organisms initially formed via simple multicellular structures [61,62].

## Data Availability

Our data are provided in electronic supplementary material [63]. We simulated the model by using Python. The code for the simulations of our model and the scripts to reproduce our figures are available in the repository: https://github.com/YuanxiaoGao/Evolution_of_reproductive_strategies_in_incipient_multicellularity.
